# Evolution of National Influenza Vaccine Platform: From Comprehensive Preclinical Safety Validation of Trivalent Influenza Vaccine to Streamlined Immunogenicity of Quadrivalent Formulation

**DOI:** 10.3390/vaccines14060477

**Published:** 2026-05-28

**Authors:** Luka Dragačević, Veljko Blagojević, Marko Vasić, Rajna Minić, Ivana Ćuruvija, Ivana Prijić, Raisa Petrović, Darko Rogić, Irena Živković

**Affiliations:** Institute of Virology, Vaccines and Sera “Torlak”, Vojvode Stepe 458, 11000 Belgrade, Serbia; vblagojevic@torlak.rs (V.B.); mvasic@torlak.rs (M.V.); rminic@torlak.rs (R.M.); iprijic@torlak.rs (I.P.); drogic@torlak.rs (D.R.);

**Keywords:** influenza vaccine, vaccine manufacturing platform, technology transfer, preclinical evaluation, pandemic preparedness

## Abstract

**Background:** The Global Action Plan for Influenza Vaccines, launched by the World Health Organization in 2006, aimed to expand global manufacturing capacity, ensure equitable access to influenza vaccines, and reduce global vaccine shortages. The Institute of Virology, Vaccines and Sera “Torlak” was recognized as a partner and participated in a technology transfer programme to improve national production capabilities. Preclinical studies conducted in 2013 and 2014 provided comprehensive toxicological evidence supporting the safety of the trivalent influenza vaccine manufacturing platform. Nearly a decade later, this validated platform enabled the independent development of a quadrivalent influenza vaccine. Subsequent preclinical studies conducted in accordance with European Medicines Agency guidelines demonstrated preserved immunogenicity despite the inclusion of an additional antigen. **Methods:** Preclinical evaluation included standard safety and immunogenicity assessments in animal models. Safety assessment included evaluation of systemic parameters (general health status, leukocyte profile, blood biochemistry, and histopathology) and local reactions. Immunogenicity of both trivalent and quadrivalent formulations was assessed using haemagglutination inhibition and microneutralization assays. **Results:** No significant systemic or local adverse effects were observed. Both trivalent and quadrivalent formulations induced robust immune responses, with immunogenicity comparable to that of corresponding commercial vaccines. **Conclusions:** These findings confirm the safety profile and demonstrate strong immunogenicity for both the trivalent and quadrivalent formulations, supporting the successful establishment of a national influenza vaccine platform, contributing to increased pandemic preparedness and sustainable influenza vaccine production.

## 1. Introduction

Scientists estimate that annual influenza epidemics results in up to 650,000 respiratory deaths worldwide, with the greatest burden observed among people aged 65 and older, children younger than 5 years, and individuals with chronic underlying conditions [1]. In addition to previously addressed direct health impacts, epidemics of influenza put significant pressure on healthcare systems and lead to economic losses through absenteeism and reduced productivity [2]. Seasonal influenza vaccination therefore remains a cornerstone of prevention strategies and remains the most effective intervention to mitigate the clinical and societal impact of seasonal influenza outbreaks. Regardless of the proven effectiveness of influenza vaccines, global access has historically been uneven and limited by manufacturing capacity, geographic concentration of production facilities and dependence on complex international supply chains. These limitations are particularly evident during pandemic scenarios, when global demand rapidly exceeds supply and competition for vaccine doses intensifies [3]. Concerns regarding pandemic preparedness intensified following the emergence of highly pathogenic avian influenza A/H5N1 in 1997, which demonstrated a high case fatality rate and potential for zoonotic transmission to humans. In response, the World Health Assembly in 2005 called for strengthened pandemic preparedness and increased investment in the research and development of new and improved influenza vaccines, including strategies to enhance production capacity and reduce antigen requirements per dose [4].

To address these challenges, the World Health Organization (WHO) launched the Global Action Plan for Influenza Vaccines (GAP) in 2006, a coordinated initiative aimed at expanding global vaccine production capacity, particularly in low- and middle-income countries, and improving equitable access to the vaccines [5]. The programme focused on increasing seasonal influenza vaccine uptake, modernizing manufacturing infrastructure, and supporting the development of more effective influenza vaccines. The 2009 H1N1 influenza pandemic further highlighted global vulnerabilities, particularly the lack of domestic production capacity and delays in vaccine availability in many regions [6].

The Institute of Virology, Vaccines and Sera “Torlak” was selected as a partner and participated in the WHO GAP technology transfer programme [4]. This collaboration enabled significant modernization of its influenza vaccine manufacturing platform following Good Manufacturing Practice standards (GMP) and supported the development of an egg-based, split, trivalent inactivated influenza vaccine (TIV). As part of the development programme, extensive preclinical safety studies were conducted in accordance with regulatory requirements at the time.

Traditionally, most licenced seasonal influenza vaccines were formulated as TIVs, containing two influenza A subtypes (A/H1N1 and A/H3N2) and a single B influenza lineage. However, the co-circulation of two antigenically distinct B influenza lineages (Victoria and Yamagata) has led to frequent mismatches between vaccine strains and circulating viruses, reducing vaccine effectiveness [7]. These epidemiological observations provided the scientific rationale for the development and introduction of quadrivalent influenza vaccines (QIVs), which include antigens from both influenza B lineages. QIVs offer broader protection, while maintaining safety and immunogenicity profiles comparable to TIVs [8]. Even though transition from TIVs to QIVs increases manufacturing complexity, manufacturing time and antigen content, it does not fundamentally alter the basic production platform.

During production, vaccine viruses were propagated in 11-day-old embryonated chicken eggs under strain-specific controlled conditions. After the harvesting of allantoic fluid, the material underwent clarification, concentration, purification, and sterile filtration. Virion splitting was performed using Triton X-100, followed by viral inactivation with β-propiolactone. Monovalent bulks were subsequently formulated into final vaccine compositions, and antigen content was determined using the single radial immunodiffusion (SRID) assay, the standard method for influenza vaccine potency testing. The manufacturing process, analytical methods, and quality controls were validated and consistently applied for both trivalent and quadrivalent formulations in accordance with relevant regulatory and pharmacopeial requirements [9,10,11]. Although the B/Yamagata lineage has not been detected globally since 2020 and its removal from seasonal influenza vaccine compositions has recently been recommended by the WHO, the transition from TIVs to QIVs remains scientifically and strategically relevant. In the present study, the development of the QIV is presented not only as a response to the epidemiological circumstances existing at the time of its introduction, but also as evidence of the successful establishment, long-term maintenance, and adaptability of a national influenza vaccine manufacturing platform. The ability to expand a validated production process toward more complex multivalent formulations demonstrates technological robustness, regulatory maturity, and preparedness for future influenza epidemiology changes or the emergence of novel strains requiring rapid vaccine adaptation.

Historically, vaccine development followed a paradigm in which any change in formulation required comprehensive preclinical reassessment, including full toxicological testing, regardless of prior experience with similar products. However, advances in regulatory science and accumulated experience with licenced influenza vaccines have led to a more flexible, risk-based approach to preclinical evaluation. Regulatory authorities now recognize the concept of platform-based vaccine development, whereby extensive prior knowledge of the manufacturing process and safety profiles can be leveraged to support incremental product modifications without unnecessary repetition of animal studies [12]. This approach is also aligned with the principles of Replacement, Reduction, and Refinement (3Rs) in animal research while preserving high safety standards.

Current European Medicines Agency (EMA) guidelines emphasize that preclinical evaluation of seasonal influenza vaccines produced using well-established egg-based platforms should be tailored to the nature and extent of the introduced modification [13]. When manufacturing processes remain unchanged and only antigen composition is expanded, a streamlined preclinical strategy may be limited to immunogenicity assessment, in line with EMA and WHO guidance. Comparability is demonstrated by the fact that the investigational vaccine was developed using the same manufacturing platform and formulation principles as a TIV already registered by the National Regulatory Authority, with the only modification being the inclusion of an additional influenza B strain. This approach is supported by EMA guidance stating that “new vaccines include those which are similar to an existing vaccine in terms of the types of antigens and anticipated interaction with the immune system (e.g., quadrivalent inactivated influenza vaccines that are manufactured similarly to trivalent inactivated vaccines)” and that “for new vaccines that have similar manufacturing processes to already authorized vaccines, nonclinical toxicology studies need not be repeated” [13]. In addition, WHO guidelines state that “for whole virion, split or subunit inactivated human influenza vaccines manufactured from an established production process and formulated similarly to a licensed seasonal influenza vaccine (apart from the strain), nonclinical safety investigations need not be repeated, provided that they have been performed in accordance with relevant WHO and national/regional requirements” [9,14].

This regulatory flexibility is important for domestic manufacturers aiming to modernize their vaccine portfolios while maintaining continuity of supply and ensuring vaccine independence in the event of a pandemic.

This study aimed to present the evolution of the influenza production process established through the WHO GAP technology transfer program, from comprehensive preclinical safety evaluation of TIVs to streamlined immunogenicity assessment of QIVs. In this context, the study demonstrates the successful application of the vaccine production platform for the development of new vaccines.

## 2. Materials and Methods

### 2.1. Materials

All materials were obtained from Merck KGaA (Darmstadt, Germany) unless otherwise stated.

### 2.2. Test Formulations

The influenza vaccine, designated TorVaxFlu, is an inactivated, split-virion trivalent vaccine manufactured by the Institute of Virology, Vaccines and Sera “Torlak” (Serbia) for adult use (18+) for the prevention of seasonal influenza. The vaccine was formulated with viral strains recommended by the World Health Organization for the influenza season 2013/2014 and was produced in facilities compliant with GMP standards [15]. The vaccine formulation contains 15 µg of hemagglutinin (HA) from each of the three strains, namely A/California/7/2009 (H1N1) pdm09-like, A/Texas/50/2012, which is an A(H3N2)virus antigenically like the cell-propagated prototype virus A/Victoria/361/2011, and B/Massachusetts/2/2012-like virus, in 0.5 mL phosphate-buffered saline (PBS) pH 7.4.

The quadrivalent vaccine of the same type, TorVaxFlu Tetra (split, inactivated, egg-derived), was produced in the same facility and in an identical manner to the registered trivalent vaccine. The vaccine formulation was prepared in accordance with the WHO recommendations for the 2022/2023 influenza season in the Northern Hemisphere and contained 15 µg of HA from each of the four strains per 0.5 mL dose: A/Victoria/2570/2019 (H1N1) pdm09-like virus, A/Darwin/9/2021 (H3N2)-like virus, B/Austria/1359417/2021 (B/Victoria lineage)-like virus, and B/Phuket/3073/2013 (B/Yamagata lineage)-like virus.

### 2.3. Animal Immunization in Safety Studies

The vaccine safety study comprised an evaluation of acute toxicity (reactions occurring within 24 h post-immunization), as well as toxicity following single-dose and repeated-dose administration (3 weeks apart). Twenty Wistar rats (10 males and 10 females), 8–10 weeks old and weighing 180 ± 5 g, were used to assess the effects of a single dose, while an additional 20 rats (10 males and 10 females) with same characteristics were immunized to evaluate the effects of repeated doses [16,17]. Group sizes were selected based on established regulatory guidance and standard practice in preclinical toxicity studies for vaccines, which typically recommend 10 animals per sex to allow for detection of relevant systemic toxic effects [18]. Animals were assigned to experimental groups using a predefined allocation procedure based on non-systematic distribution, ensuring randomization and minimizing allocation bias. All rats were housed in groups of three animals per cage, and provided with ad libitum access to a standard pellet diet, throughout the experimental period. Animals were kept in standard atmospheric conditions at a temperature of 25 ± 5 °C and 50–60% humidity throughout the experiments with a 12 h light/day cycle. In both the single and repeated studies, animals were immunized by intramuscular (i.m.) administration of the human dose of TorVaxFlu (0.5 mL of vaccine). At the end of the evaluation period (i.e., two weeks after administration for the single-dose study and two weeks after administration of the second dose for the repeated-dose study), the rats were euthanized in accordance with ethical guidelines. A control group received an equivalent volume of PBS instead of the vaccine, administered in the same manner.

Toxicity following a single administration was evaluated through: (i) assessment of acute toxicity within 24 h of vaccine administration, and (ii) assessment of delayed toxicity for a fortnight following the administration. Acute toxicity was determined by clinical examination of the animals and the injection site during the first 24 h, with subsequent pathohistological analysis performed where and if indicated. Delayed toxicity was evaluated for a fortnight following administration on the basis of somatic parameters and the overall clinical status of the animals. Delayed toxicity was assessed a fortnight after a single dose following the same methods applied for the repeated-dose toxicity testing, as described in the following section.

Repeated-dose toxicity testing involved i.m. administration of two human doses of TorVaxFlu, with a three-week interval in between the doses. During the study period, the following parameters were monitored: body temperature, body weight, and food consumption, potential clinical signs of the disease, total leukocyte count and differential leukocyte count, and hematological analysis, as well as spontaneous behaviour.

Immediate and delayed local tolerance to the TorVaxFlu vaccine was evaluated in two New Zealand White rabbits (females, 20 weeks old, approximately 3 kg). The study was conducted in accordance with the applicable guidelines at the time [19,20]. One animal was used to monitor immediate reactions to the administrated vaccine, while the other was assigned for the assessment of delayed responses. The vaccine was administered i.m. into the *musculus longissimus dorsi*, on the right side of the body, at a dose and volume equivalent to the intended human dose (45 µg HA in 0.5 mL). PBS was administered on the contralateral side as an intra-individual control. The injection site and surrounding tissue were monitored macroscopically during and immediately following administration to detect any acute local reactions. For the evaluation of delayed local tolerance, the injection site was examined every 12 h over a period of four consecutive days for signs of: erythema, edema, ecchymosis, changes in skin colour, exudation, ulceration, necrosis, desquamation, scab formation, lesions, or other comparable signs.

Safety studies were conducted in accordance with the Guidelines for Good Laboratory Practice (GLP) [21] as attested by the GLP certificate granted by the Serbian Ministry of Health and covering a period of two years starting from 30 July 2015 to 29 June 2017. The safety studies were performed within the validity period of this GLP certificate.

In accordance with previous evidence demonstrating the safety of the trivalent TorVaxFlu product, further analysis of the safety of the TorVaxFlu Tetra quadrivalent vaccine was considered unnecessary [13].

### 2.4. Animal Immunization in Immunogenicity Studies

Prior to the evaluating TorVaxFlu vaccine immunogenicity, the optimal immunization dose for mice was determined. Small groups of Intor:Swiss mice (*n* = 5 females per group, 8–10 weeks old, and body weight 28 ± 2 g) were immunized with varying vaccine doses (10 μg HA, 1 μg HA and 0.1 μg HA, in 0.1 mL volume). All mice were housed in groups of ten animals per cage, and provided with ad libitum access to a standard pellet diet, throughout the experimental period. Animals were kept in standard atmospheric conditions at a temperature of 25 ± 5 °C and 50–60% humidity throughout the experiments with a 12 h light/day cycle.

Using an analogous approach, the optimal dose for the immunogenicity assessment of the TorVaxFlu Tetra vaccine was determined. Intor:Swiss mice (*n* = 5 animals per group) were immunized i.m. with doses of 0, 0.12, 1.2, and 12 μg HA per dose. Negative control animals received an equivalent volume of PBS. The total injection volume was 100 μL, administered as a single dose, divided equally between both quadriceps (50 µL per leg). The dose selected for immunogenicity evaluation was 12 µg HA/dose. The same dose of a licenced commercial influenza vaccine was used as a positive control throughout the immunogenicity studies.

For the main immunogenicity experiments, the immunogenicity of TorVaxFlu and TorVaxFlu Tetra influenza vaccines was assessed in larger groups of the same strain of mice, with the primary objective of collecting serum samples, which were subsequently analyzed. For both studies, three groups were formed: a group of animals receiving the newly formulated vaccine, a positive control group receiving a comparable commercial vaccine, and a negative control group receiving PBS. Each group consisted of 10 female and 10 male mice, which were allocated using a predefined procedure based on non-systematic distribution. The sample size was determined based on prior experience with this experimental model and in accordance with commonly used group sizes in murine immunogenicity studies [22,23,24]. Animals were immunized once via i.m. injection, administered as 2 × 50 µL into the quadriceps, regardless of group assignment. Negative control animals received an equivalent volume of PBS, while positive controls were immunized with the commercially available trivalent vaccine for the Northern Hemisphere 2013/2014 season (Vaxigrip, Sanofi Pasteur, Lyon, France), which, similarly to TorVaxFlu, is an egg-derived, split, and inactivated vaccine. For the evaluation of TorVaxFlu Tetra, the positive control group received the commercial quadrivalent vaccine for the 2022/2023 Northern Hemisphere season (Fluzone^®^ Quadrivalent, Sanofi Pasteur, Lyon, France). Regardless of whether the vaccine was commercially manufactured or produced by “Torlak” Institute, mice received 3 µg of HA from each included strain, per dose.

Three weeks following immunization, blood samples were collected from the retro-orbital sinus of animals under xylazine/ketamine anesthesia. Animals were subsequently euthanized in accordance with applicable ethical guidelines. Serum was separated by centrifugation, heat-inactivated at 56 °C for 30 min to inactivate complement, and stored at −20 °C until further analysis.

#### Ethics Statements

Animal procedures were performed in accordance with the European Directive 2010/63/EU on the protection of animals used for scientific purposes and its amendments, as well as the European Convention ETS 123 and its Revised Appendix A guidelines [25,26,27,28].

All experiments involving laboratory animals for the Preclinical study of TorVaxFlu vaccine were conducted in accordance with internationally accepted principles of animal welfare, within projects performed in collaboration with the WHO. The institution holds an approved Animal Welfare Assurance for Foreign Institutions (OLAW, U.S. Department of Health and Human Services), Assurance No. A5996-01.

All animal experiments for the Preclinical study of TorVaxFlu vaccine were approved by the Institutional Animal Care and Use Committee (IACUC) and by the Department for Animal Welfare and Veterinary Practice, Veterinary Directorate of Ministry of Agriculture, Forestry and Water Management of the Republic of Serbia (number 323-07-01577/2016-05/1, on 25 February 2016).

All animal experiments for the Preclinical study of TorVaxFlu Tetra vaccine were approved by the IACUC and by the Department for Animal Welfare and Veterinary Practice, Veterinary Directorate of Ministry of Agriculture, Forestry and Water Management of the Republic of Serbia, (number 323-07-12928/2022-05/1 on 29 November 2022), as stated by the Serbian Animal Welfare Law.

### 2.5. Methods in Safety Studies

Body weight, body temperature and food consumption were recorded prior to immunization, every two days during the first week post-immunization, and thereafter. All measurements were performed at the same time of day. Body temperature was measured using an RET-2/RET-2-ISO rectal probe (Physitemp Instruments, LLC, Clifton, NJ, USA). Blood samples were collected from the tail vein for the determination of: total leukocyte counts, differential leukocyte counts, and blood biochemistry parameters.

Total and differential leukocyte counts were evaluated before immunization and two days after immunization using peripheral blood smears. Blood smears were stained according to the standard May–Grünwald–Giemsa method, and nucleated cellular elements were identified based on their morphological characteristics [29].

Biochemistry parameters were analyzed from blood samples collected two days post-immunization using a semi-automated veterinary biochemical spectrophotometric analyser (Vet Evolution-Biosis, Arezzo, Italy). The evaluated parameters included: alanine aminotransferase (ALT), aspartate aminotransferase (AST), alkaline phosphatase (ALP), gamma-glutamyl transferase (GGT), blood urea nitrogen (BUN), glucose, total protein, and albumin.

All data are presented as mean values derived from 10 animals per group across four experimental groups of Wistar rats. The occurrence of clinical signs and spontaneous behaviour was monitored daily throughout the experiment.

Post-mortem examination comprised a macroscopic inspection, with particular attention to the presence of any lesions, as well as changes in organ size, shape, colour, or texture, and the occurrence of any masses or other deviations from normal physiological appearance. Absolute organ weights were recorded for the kidneys, liver, lungs, spleen and quadriceps muscle. Relative organ weight was calculated as the ratio of organ weight to the final body weight of the animal at the time of sacrifice, according to the following formula: relative organ weight (%) = (organ weight/body weight) × 100. Where deviations or lesions were observed, tissue samples were collected from the affected sites for subsequent histological analysis. All necropsy procedures were conducted in accordance with accepted guidelines and standards, with adherence to ethical and safety regulations [30].

A key component of the safety assessment was the histopathological evaluation of selected organs. This analysis was conducted in accordance with recommendations from the EMA [13,18] and other international regulatory authorities [31], which emphasize the examination of tissues most relevant for assessing the safety profile of the investigational product.

Tissues collected at necropsy were fixed in 10% neutral buffered formalin and processed according to the standard paraffin-embedding protocol. Sections of 5 μm thickness were prepared, with three sections obtained from the apical, central, and terminal regions of each organ/tissue, as well as from additional sites considered relevant for analysis. Histological slides were stained using the routine haematoxylin and eosin (H&E) method [32]. Tissues from experimental and control groups were subsequently compared by histopathological examination. The following organs were included in the analysis: kidneys, liver, lungs, spleen and quadriceps muscle at the site of vaccine administration. Histopathological evaluation was conducted independently by three qualified pathologists under blinded conditions. All tissue samples were anonymized and coded prior to analysis, ensuring that the reviewers were unaware of group allocation.

Additionally, tissue samples were collected from the vaccine injection site in rabbits and processed as described above for histopathological evaluation.

### 2.6. Methods in Immunogenicity Studies

The immunogenicity studies were performed in line with EMA recommendations [13]. Vaccine-induced responses were measured using haemagglutination inhibition (HI) and microneutralization (MN) assays and results were expressed as geometric mean titres (GMT) with 95% confidence intervals, with immunogenicity assessed relative to a commercial reference vaccine. In addition to humoral immunity, cellular immune responses were assessed by detecting cytokine-secreting cells using the ELISpot assay.

#### 2.6.1. Haemagglutination Inhibition Assay

Sera samples, from which nonspecific agglutinins have been removed and diluted with receptor-destroying enzyme (RDE) solution 10 times. After that it was serially double diluted in PBS, and 25 μL was added to the wells of a V-bottom microtitre plate. Equal volumes of influenza virus at a defined titre were added to each well, followed by the addition of 50 μL 0.5% turkey red blood cells. Plates were incubated at room temperature (RT), and the HI titre was determined as the highest serum dilution that completely inhibited haemagglutination [33].

#### 2.6.2. Microneutralization Assay

To determine neutralizing antibody titre, MN assay was performed according to the WHO protocol. Initially, a 50% median tissue culture infectious dose (TCID50) was determined for each virus strain included in the vaccine. Serial dilutions of receptor-destroying enzyme-treated sera were pre-incubated with a 100× TCID50 of live virus prior to the addition of Madin–Darby canine kidney (MDCK) cells. After overnight incubation, the cells were stained with crystal violet. The neutralization titre was expressed as the reciprocal of the highest dilution of serum that gave 50% neutralization of 100× TCID50 of virus in MDCK cells [33].

#### 2.6.3. ELISpot

To identify IFN-γ-secreting cells, an ELISpot assay was performed according to the manufacturer’s instructions (Mouse IFN-γ ELISpot Kit, cat. no. EL485, R&D Systems, Minneapolis, MN, USA), including the provided positive and negative assay controls. Mice were immunized i.m. with the TorVaxFlu vaccine or Vaxigrip vaccine (positive control), at a dose of 10 μg HA per dose, while negative control mice received the same volume of PBS. Each group consisted of five females. Six days after immunization, splenocytes were isolated, erythrocytes were lysed, and cell viability was assessed (only samples with viability >80% were used).

Freshly isolated splenocytes (5 × 10^5^ cells/well in 0.2 mL) were added to plates pre-coated with anti-mouse IFN-γ antibodies. Cells were stimulated with 1 μg/well of either the TorVaxFlu or Vaxigrip vaccine, or each individual strain included in the vaccine. After 48 h incubation at 37 °C in 5% CO_2_, IFN-γ-secreting cells were detected and visualized according to the manufacturer’s protocol. Spots were quantified by counting under a dissecting microscope (Olympus VMZ Stereo Microscope, Olympus Optical Co., Ltd., Tokyo, Japan).

### 2.7. Statistical Analysis

Safety and tolerability parameters, including body weight, food consumption, body temperature, total leukocyte count, leukocyte differential, serum biochemistry, and relative organ weights, were measured for each group. Data were analyzed using one-way ANOVA to compare vaccinated animals with their respective non-vaccinated controls (PBS group), while males and females were analyzed separately. Arithmetic means ± standard deviation (SD) were calculated for each parameter. A *p*-value < 0.05 was considered statistically significant.

Statistical analysis was performed using one-way ANOVA to compare the immune responses of the investigational vaccine groups with those of the positive control group immunized with a commercial influenza vaccine. Since the HI titre was below the detection limit (<10), a value of 5 was assigned for statistical analyses of the results. HI and MN titres are presented as geometric means ± 95% confidence intervals and were log_2_-transformed prior to analysis. A *p*-value < 0.05 was considered statistically significant.

## 3. Results

### 3.1. Safety Evaluation: Acute Toxicity of TorVaxFlu Influenza Vaccine

Assessment of acute toxicity revealed no adverse effects related to vaccination within 24 h post-administration. All animals remained clinically normal, with no observable behavioural or physiological abnormalities. No changes were observed in body weight and temperature, food intake, leukocyte count, biochemical parameters), relative organ weight or histopathological findings. Given the absence of clinical, behavioural, or physiological signs of acute toxicity, no triggers for histopathological evaluation were identified, and such analysis was not performed in this study. Together, these assessments allowed for evaluation of general health status, systemic toxicity, and local tissue reactogenicity following immunization.

### 3.2. Safety Evaluation: Toxicity After Single and Repeated Doses

Toxicity assessments following single and repeated doses included measurements of body weight, food consumption, and body temperature, as well as evaluations of total and differential leukocyte counts, hematological parameters, unprovoked behaviour, necropsy findings, relative organ weights, and histological analysis of the kidneys, liver, lungs, spleen and the site of the application.

#### 3.2.1. Food Consumption, Body Weight and Body Temperature After Single and Repeated Doses

**Figure 1:**
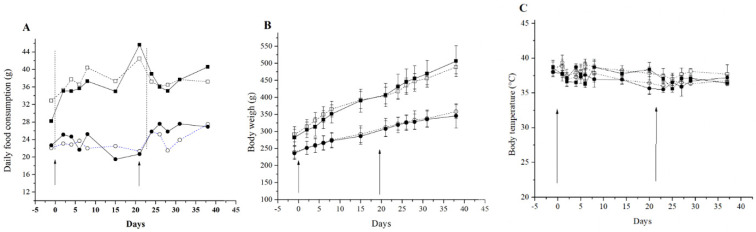
Food consumption, body weight and body temperature after a single and repeated dose of vaccine. (**A**) Food consumption, (**B**) weight gain and (**C**) body temperature measured following single- and repeated-dose vaccination. The solid lines represent immunized females (circle) and males (square); the dashed lines represent control (PBS) females (open circle) and males (open square). Arrows indicate the days of immunization. Data are presented as arithmetic mean ± SD (*n* = 10 per group). One-way ANOVA was performed to compare vaccinated and control groups. No statistically significant differences were observed.

The measured parameters food consumption, weight gain and body temperature remained unaffected after vaccine administration, as well as unprovoked behaviour occurrence, either following a single or repeated doses (Figure 1).

Clinical signs such as sternutation, nasal discharge, shivering, salivation, cough, muscle tremors, and other similar signs were not registered in either experimental group of animals.

No significant differences were observed between immunized and control animals, either following single or repeated-dose administration.

#### 3.2.2. Total and Differential Leukocyte Counts After Single and Repeated Doses

The total and differential leukocyte counts were assessed to evaluate the immune response and potential hematological effects following vaccination.

**Figure 2:**
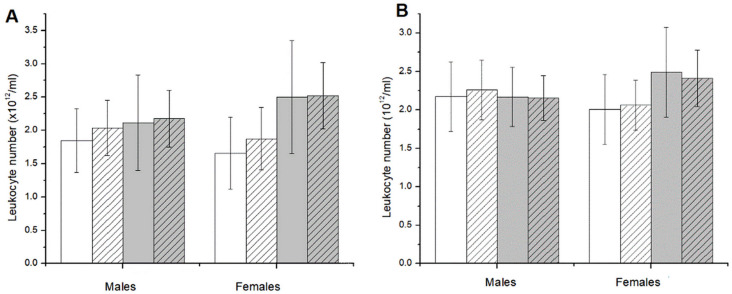
Total leukocyte counts following single (**A**) and repeated (**B**) doses of the TorVaxFlu vaccine. Groups are: white bars—PBS-treated rats; grey bars—TorVaxFlu-treated rats. Counts were evaluated before immunization (non-hatched columns) and two days after immunization (hatched columns) using peripheral blood smears. Data are presented as arithmetic mean ± SD (*n* = 10 per group). One-way ANOVA was performed to compare vaccinated and control groups. No statistically significant differences were observed.

**Figure 3:**
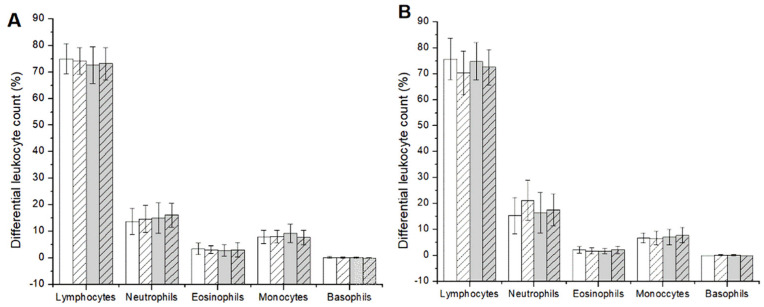
Differential leukocyte count after single dose, in females (**A**) and males (**B**). Groups are: white bars—PBS-treated rats; grey bars—TorVaxFlu-treated rats. Empty bars represent measurements before administration, while hatched bars show measurements two days after administration. Data are presented as arithmetic mean ± SD (*n* = 10 per group). One-way ANOVA was performed to compare vaccinated and control groups. No statistically significant differences were observed between vaccinated and control groups.

**Figure 4:**
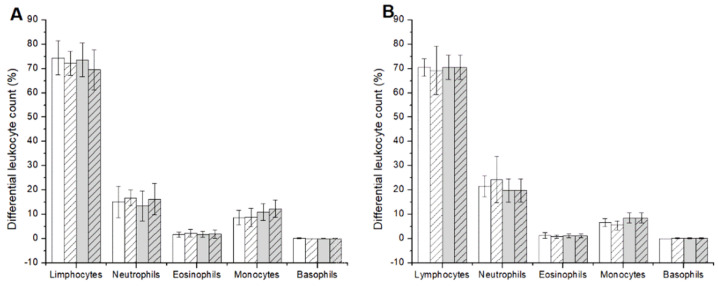
Differential leukocyte counts after repeated doses, in females (**A**) and males (**B**). Groups are: white bars—PBS-treated rats; grey bars—TorVaxFlu-treated rats. Empty bars represent measurements before administration, while hatched bars show measurements two days after administration. Data are presented as arithmetic mean ± SD (*n* = 10 per group). One-way ANOVA was performed to compare vaccinated and control groups. No statistically significant differences were observed between vaccinated and control groups.

The total (Figure 2) and differential leukocyte counts (Figure 3 and Figure 4) did not differ significantly between vaccinated and control rats, regardless of whether animals received a single or double vaccine dose, indicating that vaccination had no measurable impact on hematological parameters. The observed leukocyte counts were as expected, as leucocytosis is usually seen with live attenuated or adjuvant vaccines, where inactivated influenza vaccines do not significantly alter total leukocyte counts. Nevertheless, these vaccines effectively stimulate lymphocyte activation and antibody production. Consistent with this, no significant changes in leukocyte differential counts were observed in vaccinated mice compared to controls, indicating that the vaccine induced an immune response without affecting overall leukocyte distribution.

#### 3.2.3. Blood Biochemistry After Single and Repeated Doses

Blood biochemistry was analyzed following a single dose (Table 1) and repeated doses (Table 2) of the vaccine to evaluate any potential impact on biochemical parameters and metabolic function. Each analyzed biochemical parameter was evaluated using samples obtained from 10 animals in each experimental group.

Biochemical analysis of serum samples from Wistar rats demonstrated that all measured parameters (ALT, AST, ALP, GGT, BUN, glucose, total protein, and albumin) remained within the physiological reference ranges for this strain. Comparisons between vaccinated and control animals within each sex revealed no significant differences, indicating that the vaccine did not induce alterations in these parameters. Some differences were observed between males and females, such as slightly higher ALT, AST, BUN, and total protein in males, which are consistent with known sex-related physiological variations and are not attributable to vaccination.

#### 3.2.4. Relative Organ Weights After Single and Repeated Doses

Necropsy is a crucial procedure in toxicology studies, as it enables the comprehensive assessment of organ changes which can indicate potential toxic effects. Relative organ weight represents an integrated parameter reflecting treatment-related effects on specific organs while accounting for differences in overall body weight, and is therefore widely used as a sensitive indicator of systemic toxicity [34]. Particular emphasis is placed on organs such as the thymus, owing to its highly stress-sensitive involution; the liver, as changes are commonly associated with exposure to toxic substances; the heart, where variations in organ weight may indicate potential cardiotoxicity; and the kidneys, in which weight alternations are considered indicative of renal toxicity.

The survival rate of Wistar rats in this study was 100%. At the time of scheduled sacrifice, a systematic macroscopic necropsy examination was performed on all rats. No treatment-related macroscopic abnormalities were observed in any of the examined organs. All organs exhibited normal size, shape, colour, consistency, and anatomical situs, with no evidence of lesions, congestion, edema, hemorrhage, adhesions, or other pathological findings. The necropsy findings were comparable between immunized and control groups following both single-dose and repeated-dose administration.

**Figure 5:**
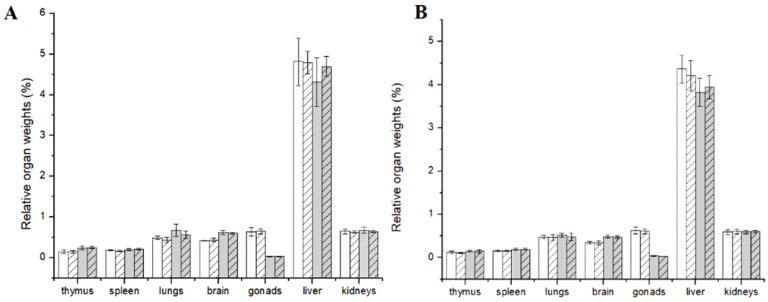
Relative organ weights of Wistar rats three weeks after (**A**) a single dose (45 µg HA per dose) or (**B**) a second dose (2 × 45 µg HA per dose) of the split influenza vaccine. Groups are: white bars—male rats; grey bars—female rats. Empty bars represent PBS-treated rats, while hatched bars show TorVaxFlu-treated rats. Data are presented as arithmetic mean ± SD (*n* = 10 per group). One-way ANOVA was performed to compare vaccinated and control groups. No statistically significant differences were observed between vaccinated and control groups.

Following administration of a single vaccine dose as well as repeated doses (Figure 5), both absolute and relative organ weights (expressed as organ-to-body weight ratios) of all examined organs in immunized animals remained unchanged compared to control animals.

#### 3.2.5. Histopathological Findings Following Single and Repeated Immunizations

**Figure 6:**
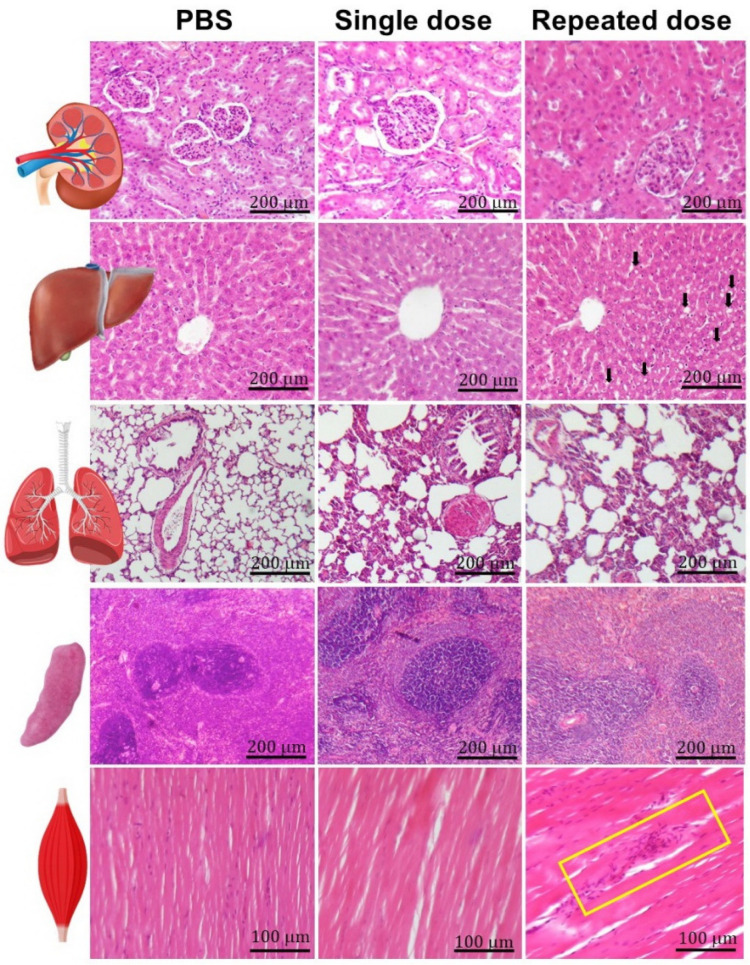
Histological sections of rat kidney, liver, lung, spleen, and injection site tissues after administration of the PBS, single dose, and repeated dose. Tissues were analyzed for histopathological changes. The results of this analysis revealed no significant changes, suggesting that the vaccine does not induce harmful effects on these organ tissues, further indicating its safety in terms of systemic toxicity. As expected, a mild local reaction was observed at the injection site, which is typical for i.m. vaccines and does not indicate any severe adverse reactions. Black arrows mark hepatocytes within the liver parenchyma that exhibited prominent cytoplasmic vacuoles displacing the nuclei peripherally. Yellow rectangle shows monocyte–macrophage inflammatory cells.

In the PBS, single-dose, and repeated-dose rat groups, the architecture of the kidney, lung, spleen and quadriceps muscle tissues was preserved, with no evidence of degenerative changes or inflammation (Figure 6). In the repeated-dose group, hepatocytes within the liver parenchyma exhibited prominent cytoplasmic vacuoles displacing the nuclei peripherally (arrows). The vacuoles observed in hepatocytes may indicate lipidosis, a common response to mild toxic or stress factors, but do not signify severe damage. Their presence in the repeated-dose group suggests some hepatic changes, though no significant degenerative or inflammatory alterations were observed. In addition, quadriceps muscle at the injection site demonstrated multifocal infiltration by monocyte–macrophage inflammatory cells (yellow rectangle).

#### 3.2.6. Immediate and Delayed Local Reaction Following Immunizations

**Figure 7:**
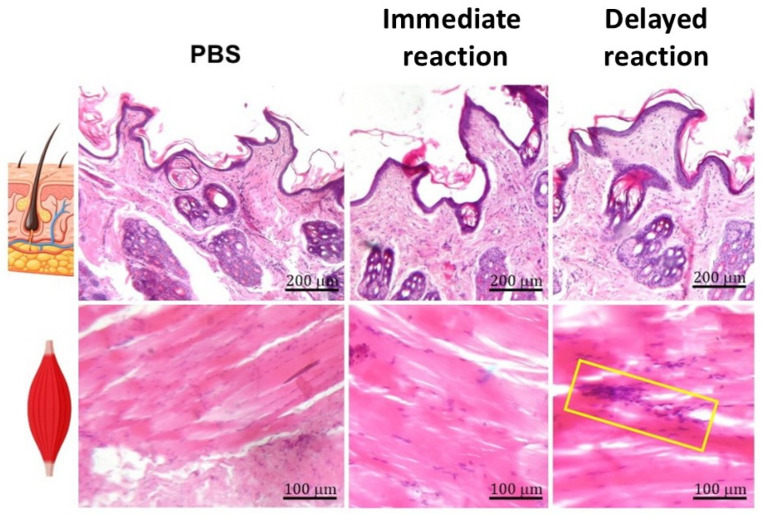
Histological sections of rabbit skin and injection site tissues after administration of the PBS and immediate and delayed reaction after vaccine administration. Tissues were analyzed for histopathological changes. Yellow rectangle marks multifocal areas of monocyte–macrophage inflammatory cell infiltration.

In the rabbit evaluated for immediate local tolerance, the architecture of the skin and underlying muscle tissue was preserved at both the vaccine and PBS injection sites, with no evidence of degenerative changes or inflammation (Figure 7).

In the rabbit evaluated for delayed local tolerance at the vaccine injection site, multifocal areas of monocyte–macrophage inflammatory cell infiltration were observed (yellow rectangle). This finding was also evident in the injection site analysis of rats, confirming a similar inflammatory response. Such transient local inflammation is a typical early response to i.m. vaccine administration and is generally not associated with adverse effects.

Results obtained during safety evaluation of toxicity after single and repeated doses are within the clinical health indicators, which are key parameters for demonstrating vaccine safety (Figure 1, Figure 2, Figure 3, Figure 4 and Figure 5; Table 1 and Table 2). These observations are consistent with the animals’ unaltered external appearance and normal behaviour throughout the experiment, which represent important, although often overlooked, indicators of the general condition of laboratory animals. Overall, these findings suggest that the vaccine did not induce detectable adverse effects.

### 3.3. Evaluation of Immunogenicity of the TorVaxFlu Influenza Vaccine

The optimal immunization dose used to evaluate vaccine immunogenicity was determined in a preliminary dose-finding study. Mice were immunized with vaccine doses ranging from 0.1 to 10 µg per dose (0.1, 1, and 10 µg), and sera collected from these animals were analyzed using the HI assay. Based on the HI antibody responses obtained (Supplementary Table S1), the dose inducing the most robust immune response (10 µg per dose) was selected for subsequent immunization experiments and used as the reference dose for assessing vaccine immunogenicity.

#### 3.3.1. HI Antibody Responses in Sera of Mice Immunized with the TorVaxFlu TIV

**Figure 8:**
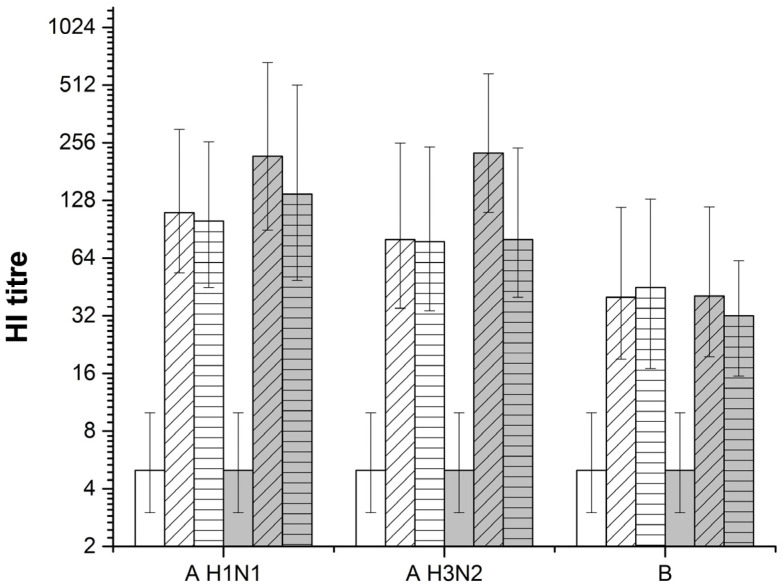
HI titres against three influenza strains (A/H1N1, A/H3N2, B) measured individually in mice sera following immunization. For each strain, columns represent: male PBS group (white bars), female PBS group (grey bars), male TorVaxFlu group (white bars with diagonal hatching), female TorVaxFlu group (grey bars with diagonal hatching), male positive control vaccine group (white bars with horizontal hatching), and female positive control vaccine group (grey bars with horizontal hatching). The dashed line indicates the limit of detection (LOD). Serum titres are expressed as GMT with 95% CI (*n* = 10 per group). Statistical comparisons were made using one-way ANOVA. No statistically significant differences were observed compared with the positive control group.

The hemagglutination inhibition (HI) assay, considered the gold standard for the evaluation of influenza vaccine immunogenicity, was used to indirectly assess the induction of functional antibody responses following immunization (Figure 8). Mice immunized with the experimental trivalent vaccine, TorVaxFlu, developed significantly higher HI antibody titres compared with the PBS-treated control group.

Importantly, the magnitude of the HI response induced by the experimental vaccine was comparable to that elicited by the licenced commercial influenza vaccine used as a positive control, as no statistically significant differences were observed between the two vaccinated groups. In addition, no gender-related differences in antibody responses were detected.

These findings demonstrate that the TorVaxFlu vaccine effectively induces a robust humoral immune response with functional antibody activity comparable to that achieved by the licenced influenza vaccine.

#### 3.3.2. MN Antibody Responses in Sera of Mice Immunized with the TorVaxFlu TIV

**Figure 9:**
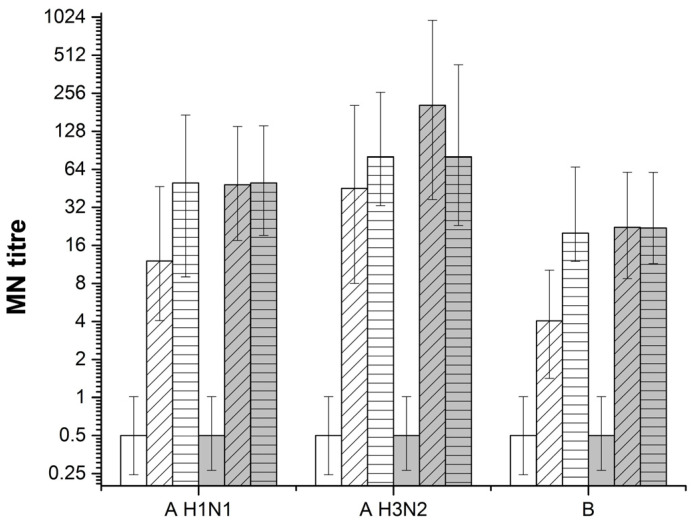
MN antibody titres in sera of mice immunized with the TorVaxFlu vaccine. For each strain, columns represent: male PBS group (white bars), female PBS group (grey bars), male TorVaxFlu group (white bars with diagonal hatching), female TorVaxFlu group (grey bars with diagonal hatching), male positive control vaccine group (white bars with horizontal hatching), and female positive control vaccine group (grey bars with horizontal hatching). Data are presented as GMT with 95% CI (*n* = 10 per group). Statistical comparisons were made using one-way ANOVA. No statistically significant differences were observed compared with the positive control group.

Vaccine-induced antibody responses in mice were measured by MN assay to determine neutralizing antibody titres in sera collected from mice immunized with the TorVaxFlu TIV (Figure 9). The MN assay demonstrated that sera from vaccinated mice contained antibodies capable of directly neutralizing live influenza virus, thereby confirming the functional activity of the induced antibodies.

Neutralizing antibody levels induced by the experimental vaccine were comparable to those elicited by the positive control (commercial) influenza vaccine, with no statistically significant differences observed between the groups, although equal vaccine doses were administered to animals in both groups.

These findings are consistent with the HI assay results and support the conclusion that the experimental vaccine induces a potent and functional humoral immune response.

#### 3.3.3. IFN-γ-Producing Cellular Immune Response Induced by the Trivalent Influenza Vaccine

**Figure 10:**
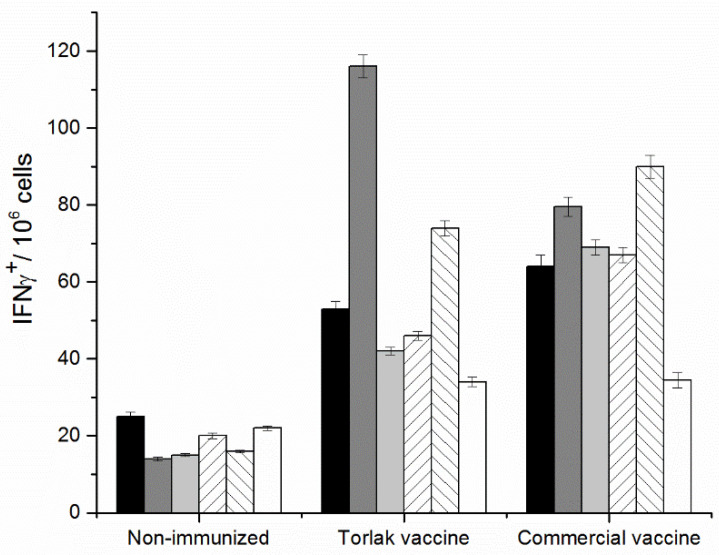
IFN-γ ELISpot assay results obtained from splenocytes of mice immunized with the TorVaxFlu vaccine, the Vaxigrip vaccine (positive control), or PBS alone (negative control). The number of IFN-γ-producing splenocytes is shown as spot-forming cells per 10^6^ splenocytes following in vitro stimulation with: medium alone (white bars), individual influenza vaccine antigens (A/H1N1—black bars; A/H3N2—dark grey bars; B—light grey bars), the TorVaxFlu vaccine (upward-diagonal hatched bars), or the Vaxigrip vaccine (downward-diagonal hatched bars). Data are presented as arithmetic mean ± SD. Statistical comparisons between vaccinated and control groups were performed using one-way ANOVA. No statistically significant differences were observed.

Beyond confirming the successful induction of a humoral immune response, with both indirect, HI, and direct, MN, evidence of its functional activity, we also assessed the induction of a vaccine-driven cellular immune response (Figure 10). This response was primarily characterized by IFN-γ secretion, reflecting vaccine-induced Th1 effector activation relevant for antiviral defence.

Six days after immunization with the trivalent influenza vaccine, mice were sacrificed and splenic lymphocytes were isolated. The lymphocytes were then stimulated in vitro with each individual vaccine antigen, as well as with the investigational (TorVaxFlu) or the positive control influenza vaccine. Antigen-specific IFN-γ-secreting cells, a key cytokine involved in Th1-biassed immune responses and support of antibody production, were detected and quantified using the ELISpot assay. Although some variation in the number of IFN-γ-secreting cells was observed between the TorVaxFlu and positive control vaccine groups, these differences were not statistically significant (Figure 10). These findings indicate that all vaccine components were capable of inducing a Th1-oriented cellular response associated with antiviral immunity.

### 3.4. Evaluation of Immunogenicity of the TorVaxFlu Tetra Influenza Vaccine

The optimal immunization dose used to evaluate vaccine immunogenicity of the quadrivalent influenza vaccine was determined in a preliminary dose-finding study. Mice were immunized with varying antigen concentrations and antibody responses were evaluated using the HI assay, as performed for the trivalent vaccine. HI titres were measured in sera of Intor:Swiss mice following immunization with the quadrivalent split influenza vaccine for the 2022/2023 season at the following HA doses per mouse: 0 μg, 0.12 μg, 1.2 μg, and 12 μg (Supplementary Table S2). A commercial influenza vaccine administered at 12 μg HA per dose was included as a positive control.

According to these results, the dose selected for immunization of mice in preclinical studies was 12 μg of total HA per dose, corresponding to 3 μg of HA per dose for each individual vaccine influenza strain.

#### 3.4.1. HI Antibody Responses in Sera of Mice Immunized with the TorVaxFlu Tetra QIV

**Figure 11:**
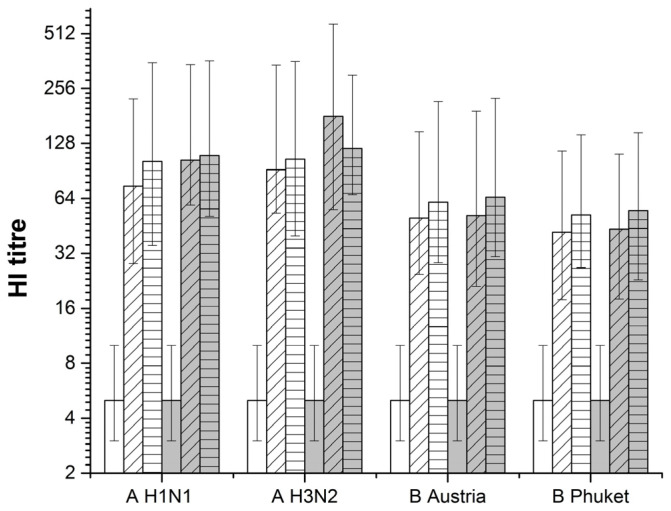
HI titres in sera of Intor:Swiss mice immunized with a QIV at a dose of 12 µg total HA per dose. For each strain, columns represent: male PBS group (white bars), female PBS group (grey bars), male TorVaxFlu group (white bars with diagonal hatching), female TorVaxFlu group (grey bars with diagonal hatching), male positive control vaccine group (white bars with horizontal hatching), and female positive control vaccine group (grey bars with horizontal hatching). The dashed line indicates the LOD. Data are presented as GMT with 95% CI ± SD (*n* = 10 per group). Statistical comparisons were performed using one-way ANOVA. No statistically significant difference was observed compared with the positive control group.

The immunogenicity of the TorVaxFlu Tetra (QIV) was evaluated using the hemagglutination inhibition (HI) assay, the standard method for assessing functional antibody responses against influenza viruses (Figure 11). Immunization with the experimental vaccine resulted in a significant increase in HI antibody titres compared with the PBS control group, confirming efficient induction of vaccine-specific humoral immunity.

The antibody responses generated by TorVaxFlu Tetra were comparable to those observed in animals immunized with the licenced commercial quadrivalent influenza vaccine, with no statistically significant differences detected between the vaccinated groups. Furthermore, analysis of antibody responses according to sex revealed no significant gender-associated variations.

Overall, these results indicate that the TorVaxFlu Tetra vaccine induces a strong and functional humoral immune response comparable to that achieved with the commercially available influenza vaccine.

#### 3.4.2. MN Antibody Responses in Sera of Mice Immunized with the TorVaxFlu Tetra QIV

MN assays performed using sera from immunized mice detected functional antibodies capable of recognizing and neutralizing the cytopathic effects of live viruses corresponding to the vaccine strains.

**Figure 12:**
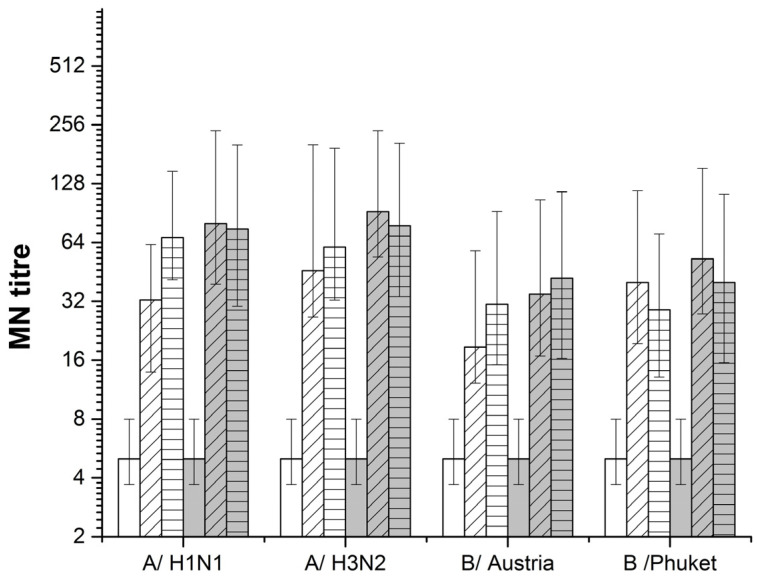
MN titres in sera of Intor:Swiss mice immunized with the QIV at a dose of 12 μg total HA per dose. For each strain, columns represent: male PBS group (white bars), female PBS group (grey bars), male TorVaxFlu group (white bars with diagonal hatching), female TorVaxFlu group (grey bars with diagonal hatching), male positive control vaccine group (white bars with horizontal hatching), and female positive control vaccine group (grey bars with horizontal hatching). Data are presented as GMT with 95% (*n* = 10 per group). Statistical comparisons were made using one-way ANOVA. No statistically significant difference was observed compared with the positive control group.

Serum samples obtained from mice immunized with the TorVaxFlu Tetra QIV were analyzed by microneutralization (MN) assay to assess the induction of functional neutralizing antibodies against influenza viruses included in the vaccine formulation (Figure 12). Vaccinated animals developed detectable neutralizing antibody responses capable of inhibiting live virus infection in vitro, demonstrating the biological functionality of vaccine-induced antibodies.

The magnitude of the neutralizing antibody response was similar to that observed in mice receiving the licenced commercial influenza vaccine used as a positive control, with no statistically significant differences detected between the experimental and control groups following administration of equivalent vaccine doses.

Overall, the MN results corroborate the findings obtained by the HI assay and indicate that the TorVaxFlu Tetra formulation effectively stimulates a strong functional humoral immune response against all vaccine-included influenza strains.

## 4. Discussion

The present study demonstrates that both the trivalent and quadrivalent influenza vaccines developed by the Institute of Virology, Vaccines and Sera “Torlak” exhibit favourable safety and immunogenicity profiles in preclinical models. These results support the validity of the underlying egg-based, split, inactivated platform, obtained during GAP technology transfer, and should be interpreted within the context of platform-based vaccine development, where certain modifications (such as inclusion of an additional B antigen) are evaluated based on prior knowledge of the manufacturing process and established safety profiles.

The streamlined nonclinical strategy applied in this study is consistent with current EMA and WHO recommendations for seasonal influenza vaccines produced using established manufacturing platforms. Both international regulatory authorities recognize that, when vaccine composition changes are limited to strain updates or expansion of antigen composition without modification of the manufacturing process, formulation principles, or mechanism of action, extensive repeated toxicological testing may not provide additional scientific value. EMA guidance emphasizes that nonclinical evaluation should follow a risk-based approach, taking into account prior knowledge of the platform, manufacturing consistency, and existing safety data, while WHO recommendations similarly support reduced nonclinical requirements for inactivated influenza vaccines produced using previously validated processes [9,13,14]. In this context, the present study demonstrates how accumulated platform knowledge and historical preclinical data can support efficient vaccine development while maintaining appropriate standards of safety and immunogenicity assessment.

The obtained results in this study are in accordance with the regulatory concept that extensive nonclinical toxicology testing may not be necessary when modifying currently developed formulations using a well-established platform. By adopting this kind of framework, it not only saves resources but also eliminates the need for additional laboratory animal use.

Taking the above into account, a key aspect of this study was to evaluate immunogenicity, safety, and local tolerability for quadrivalent formulation, in a manner analogous to that previously undertaken for the trivalent formulation and to demonstrate the comparability of the Institute “Torlak” vaccines with commercially available, licenced products, manufactured on an egg-based platform using a similar production process. The trivalent formulation was compared to Vaxigrip, while the quadrivalent formulation was compared to Fluzone, both vaccines manufactured by Sanofi Pasteur (France).

Acute, single-dose, and repeated-dose toxicity studies were conducted using i.m. administration of a full human dose of the “Torlak” influenza vaccine, in accordance with WHO and EMA guidelines [9,10,11,16,17,18], which are commonly followed by other researchers in the field for such studies [35]. These results showed no significant or visible changes, indicating the absence of toxic effects. Based on these findings, the TorVaxFlu influenza vaccine demonstrated safety in our animal model.

From the local tolerance study conducted in rabbits and rats, it can be concluded that a single i.m. administration of a full human dose of TorVaxFlu vaccine did not induce any immediate or delayed harmful local reaction at the injection site. Overall, these observations align with the expected profile of inactivated influenza vaccines, reinforcing a science-driven, risk-based approach to preclinical evaluation and further confirming the safety of the TorVaxFlu vaccine.

The immunogenicity evaluation confirms that the influenza vaccines developed by the Torlak Institute are capable of inducing both humoral and cellular immune responses. Vaccination induced neutralizing specific to the viral antigens present in the vaccines. Their functional capacity against these influenza strains was demonstrated through both the HI assay, an indirect functional method, and the MN assay, a direct functional method. In parallel, the vaccine stimulated cellular immunity, as evidenced by IFN-γ synthesis, highlighting its ability to elicit a balanced Th1/Th2 response.

From the immunogenicity studies performed in mice, it can be concluded that a single i.m. administration of 10 µg HA/dose of the TorVaxFlu vaccine, i.e., 12 µg HA/dose of the TorVaxFlu Tetra vaccine, induced an immunological humoral response directed against the vaccinal strains, compared to negative control animals as measured by HA and MN. When compared to the positive control antisera (trivalent Vaxigrip or quadrivalent Fluzone), the TorVaxFlu and TorVaxFlu Tetra, respectively, elicited an immune response of similar magnitude. When compared with alternative influenza vaccine production platforms, including cell culture-based and recombinant technologies, the egg-based approach used in this study remains highly relevant. While newer platforms offer advantages, such as shorter production timelines, avoidance of egg-adaptive mutations and independence from embryonated egg supply, the egg-based approach’s extensive production experience and global infrastructure, long-standing regulatory acceptance and well-established safety profile enables reliable large-scale supply.

Furthermore, its proven scalability for seasonal vaccine production and compatibility with established quality control methods support transition from trivalent to quadrivalent formulation without major process modifications. In addition, rapid scale-up during pandemics may be more constrained for newer platforms. Continued optimization of egg-based systems, combined with their reliability and global accessibility, ensures their ongoing importance in influenza vaccine production, particularly where alternative technologies remain limited. The study demonstrates how an existing production platform can be adapted to more complex multivalent formulations while maintaining safety, immunogenicity, and regulatory comparability. These findings provide practical evidence supporting platform sustainability, technology transfer success, and manufacturing preparedness for future epidemiological changes, including the potential emergence of novel influenza variants or other vaccine targets requiring rapid adaptation.

From a broader perspective, the findings of this study have important implications for pandemic preparedness and global vaccine equity. The ability to leverage a validated manufacturing platform to rapidly develop updated vaccine formulations enhances responsiveness to evolving epidemiological needs. This flexibility is particularly relevant for influenza, where both seasonal variability and pandemic threats require adaptable production strategies. Furthermore, the successful development of a quadrivalent vaccine within a national manufacturing framework highlights the importance of strengthening domestic production capacity. Global disparities in vaccine access, observed during both influenza pandemics and the COVID-19 pandemic, underscore the need for regional and national self-sufficiency.

Technology transfer initiatives and sustainable local production platforms, such as the one described, represent key components in reducing dependence on global supply chains and ensuring more equitable access to vaccines.

## 5. Conclusions

Our studies have demonstrated that both TIVs and QIVs can be successfully produced on a new manufacturing platform and have shown that both are safe and immunogenic. These findings not only confirm the feasibility of developing effective vaccines but also underscore their potential to strengthen domestic public health and reduce reliance on global supply chains during seasonal epidemics. By expanding domestic production capacity, it builds independence from external manufacturers and increases the level of national security. Furthermore, it is crucial that, in the event of a pandemic, this well-validated production platform can contribute to global preparedness and protection efforts.

## Figures and Tables

**Table 1:** Blood biochemistry parameters after a single dose.

Biochemical Parameter/Sex	Vaccine/Male	PBS/Male	Vaccine/Female	PBS/Female
ALP (U/L)	160 ± 10	162 ± 11	140 ± 10	142 ± 8
AST (U/L)	67 ± 5	68 ± 4	63 ± 4	61 ± 4
ALT (U/L)	27 ± 3.5	26 ± 4	25 ± 4	24 ± 3
GLU (mg/dL)	125 ± 8	127 ± 7	121 ± 7	120 ± 5
BUN (mg/dL)	21 ± 3	22 ± 2	20 ± 3	20 ± 2
TP (g/dL)	6.5 ± 0.7	6.6 ± 0.5	6.4 ± 0.6	6.5 ± 0.6
Alb (g/dL)	4.1 ± 0.4	4.2 ± 0.3	4 ± 0.3	3.9 ± 0.2

**Table 2:** Blood biochemistry parameters after a repeated dose.

Biochemical Parameter/Sex	Vaccine/Male	PBS/Male	Vaccine/Female	PBS/Female
ALP (U/L)	165 ± 9	160 ± 9	139 ± 11	144 ± 10
AST (U/L)	69 ± 7	66 ± 5	61 ± 3	60 ± 3
ALT (U/L)	25 ± 2	27 ± 3	26 ± 3	23 ± 3
GLU (mg/dL)	122 ± 10	120 ± 9	119 ± 9	122 ± 4
BUN (mg/dL)	20 ± 3	21 ± 2	21 ± 2	21 ± 2
TP (g/dL)	5.5 ± 0.5	6.5 ± 0.4	6.5 ± 0.4	6.3 ± 0.5
Alb (g/dL)	4.0 ± 0.3	4.1± 0.1	5 ± 0.4	3.7 ± 0.2

## Data Availability

The original contributions presented in this study are included in the article. Further inquiries can be directed to the corresponding author.

## Supplementary Materials

The following supporting information can be downloaded at: https://www.mdpi.com/article/10.3390/vaccines14060477/s1. Table S1: HI antibody titres in the trivalent vaccine dose-finding study; Table S2: HI antibody titres in the quadrivalent vaccine dose-finding study.

## Abbreviations

WHO	World Health Organization
GAP	Global Action Plan for Influenza Vaccines
EMA	European Medicines Agency
TIV	Trivalent influenza vaccine
QIV	Quadrivalent influenza vaccines
GMP	Good manufacturing practice
HA	Hemagglutinin
PBS	Phosphate-buffered saline
GLP	Good laboratory practice
IACUC	Institutional Animal Care and Use Committee
ALT	Alanine aminotransferase
AST	Aspartate aminotransferase
ALP	Alkaline phosphatase
GGT	Gamma-glutamyl transferase
BUN	Blood urea nitrogen
H&E	Haematoxylin and eosin
HI	Inhibition hemagglutination
LOD	Limit of detection
MN	Microneutralization
RDE	Receptor-destroying enzyme
RT	Room temperature
TCID	Tissue culture infectious dose
