# Supplementary material for: Evolution of National Influenza Vaccine Platform: From Comprehensive Preclinical Safety Validation of Trivalent Influenza Vaccine to Streamlined Immunogenicity of Quadrivalent Formulation
Source: Vaccines (Basel). 2026 May 28;14(6):477. doi: 10.3390/vaccines14060477 (PMC13307876; doi:10.3390/vaccines14060477)
Supplement: Supplementary file 1 [file vaccines-14-00477-s001.zip › vaccines-4314396-supplementary.pdf]

Table S1. HI antibody titres in the trivalent vaccine dose-finding study

	TorVaxFlu						Positive control vaccine	
Dose / Inhibitor	0,1 µg HA/dose		1 µg HA/dose		10 µg HA/dose		10 µg HA/dose	
	GMT	95%CI	GMT	95%CI	GMT	95%CI	GMT	95%CI
A/California/7/2009 (H1N1) pdm09-like virus	33,63	2,59-6,53	52,78	4,77-6,69	211	6,14-10,32	128	4,81-9,19
A/Texas/50/2012 (H3N2)-like virus	11,89	0,66-4,48	160	4,18-10,38	80	5,52-9,12	80	4,13-8,51
B/Massachusetts/2/2012-like virus	28,28	2,85-5,77	45,9	5,05-6,61	121	5,56-9,3	32	2,81-7,19

Table S2. HI antibody titres in the quadrivalent vaccine dose-finding study

	TorVaxFlu Tetra						Positive control vaccine	
Dose / Inhibitor	0,12 µg HA/dose		1,2 µg HA/dose		12 µg HA/dose		12 µg HA/dose	
	GMT	95%CI	GMT	95%CI	GMT	95%CI	GMT	95%CI
A/Victoria/2570/2019 (H1N1) pdm09-like virus	50,08	5,42-5,36	77,23	5,54-6	110,46	6,52-8,01	105	5,36-8,1
A/Darwin/9/2021 (H3N2)-like virus	42,3	5,1-5,68	61,99	5,65-6,23	112	6,46-7,5	118	5,75-7,99
B/Austria/1359417/2021 (B/Victoria lineage)-like virus	37,66	4,05-6,41	46	4,77-6,27	18	4,77-6,27	80	5,32-7,32
B/Phuket/3073/2013 (B/Yamagata lineage)-like virus	18,65	3,34-5,10	22	3,58-5,34	35	4,27-5,99	30	4,05-5,77
